# Application of Solid Dispersion Technique to Improve Solubility and Sustain Release of Emamectin Benzoate

**DOI:** 10.3390/molecules24234315

**Published:** 2019-11-26

**Authors:** Bin Bin Huang, Dong Xu Liu, De Kun Liu, Gang Wu

**Affiliations:** Key Laboratory of Biopesticide and Chemical Biology (Ministry of Education), Fujian Agriculture and Forestry University, Fuzhou 350002, China; bbhuang@fafu.edu.cn (B.B.H.); ldx51296@163.com (D.X.L.); cola520@126.com (D.K.L.)

**Keywords:** polyvinyl pyrrolidone, polyacrylic resin, solubilization, sustained-release, insecticidal activity, stability

## Abstract

The solid dispersion technique, which is widely used in the medical field, was applied to prepare a pesticide dosage form of emamectin benzoate (EM). The preparation, physicochemical characterization, aqueous solubility, release dynamics, photolytic degradation, bioactivity, and sustained-release effects of the prepared EM solid dispersions were studied by a solvent method, using polymer materials as the carriers. Water-soluble polyvinyl pyrrolidone (PVP) K30 and water-insoluble polyacrylic resin (PR)III were used as the carriers. The influence of various parameters, such as different EM:PVP-K30 and EM:PRIII feed ratios, solvent and container choices, rotational speed and mixing time effects on pesticide loading, and the entrapment rate of the solid dispersions were investigated. The optimal conditions for the preparation of EM-PVP-K30 solid dispersions required the use of methanol and a feed ratio between 1:1 and 1:50, along with a rotational speed and mixing time of 600 rpm and 60 min, respectively. For the preparation of EM-PRIII solid dispersions, the use of methanol and a feed ratio between 1:4 and 1:50 were required, in addition to the use of a porcelain mortar for carrying out the process. Under optimized conditions, the prepared EM-PVP-K30 solid dispersions resembled potato-like, round, and irregular structures with a jagged surface. In contrast, the EM-PRIII solid dispersions were irregular solids with a microporous surface structure. The results of X-ray powder diffraction (XRD), differential scanning calorimetry (DSC), ultraviolet (UV) spectrometry, and infrared (IR) spectrometry showed that the solid dispersions were formed by intermolecular hydrogen bonding. The solid dispersion preparation in PVP-K30 significantly improved the solubility and dissolution rate of EM, particularly the aqueous solubility, which reached a maximum of 37.5-times the EM technical solubility, when the feed ratio of 1:10 was employed to prepare the dispersion. Importantly, the wettable powder of EM-PVP-K30 solid dispersion enhanced the insecticidal activity of EM against the *Plutella xylostella* larvae. Furthermore, the solid dispersion preparation in PRIII afforded a significant advantage by prolonging the EM technical release in water at a pH below 7.0, especially when the PRIII content in solid dispersions was high. While the amplified toxicity of the wettable powder of EM-PRIII solid dispersions against the *P. xylostella* larvae showed no significant differences from that of the EM technical, the long-term toxicity under the field condition was much better than that of the commercially available EM 1.5% emulsifiable concentrate. Notably, solid dispersions with both the PVP-K30 and PRIII carriers reduced the effect of UV photolysis.

## 1. Introduction

Emamectin benzoate (EM), a biosynthetic derivative of abamectin, is a highly selective and safe insecticide and acaricide that is known for its high target selectivity and safety [1,2]. EM is a γ-aminobutyric acid (GABA) and glutamate-gated chloride channel agonist and is highly effective against Lepidopteran, Dipteran, Homopteran, and Thrip pests [3]. Therefore, EM formulation is widely used across the world, particularly in China. However, significant resistance to EM in its target pests has been observed because of the excessive and long-term use of this insecticide [4]. Despite its widespread and efficacious use, EM has traditionally suffered from several drawbacks. Its light-sensitivity leads to its facile degradation upon exposure to the sun, particularly in an alkaline environment, which greatly limits its biological activity in agricultural fields [5,6]. A lot of studies have been done in order to overcome its photodegradable disadvantage by preparing sustained release formulations using carboxymethyl chitosan, cellulose acetate butyrate, polydopamine-*g*-poly(*N*-isopropylacrylamide), polylactic acid, hydroxypropyl-β-cyclodextrin, and so on to modify EM [7,8,9,10,11]. Additionally, its poor solubility further limits its applications. To circumvent these challenges, large quantities of adjuvants (for example, wetting agents) are typically added to the EM preparations to enhance its solubility. However, the improvements realized in these approaches are far from ideal. The related approach of preparing emulsifiable concentrates of EM requires the excessive use of organic solvents, which may cause environmental pollution. These two challenges, which severely limit the applicability of EM, could be solved by the application of the solid dispersion technique.

The solid dispersion technique was developed by Chiou and Riegelman [12] and is widely used in the field of medicine and is known in certain instances to improve the solubility, dissolution rate, and oral absorption of some poorly water-soluble drugs [13]. Water-soluble carriers, such as polyethylene glycols (PEGs) and polyvinyl pyrrolidones (PVPs), are the most common polymer carriers used for improving solubility in solid dispersions [14]. A large number of water-soluble carrier materials suitable for solid dispersions have emerged recently, and include hydroxypropyl cellulose (HPC), hydroxypropylmethyl cellulose (HPMC), hydroxypropylmethyl cellulose phthalate (HPMCP), chitosans, and gelucires [15,16,17,18,19]. Importantly, the application of the solid dispersion technique to water-soluble drugs using water-insoluble polymers, such as ethyl cellulose (EC) and polymethacrylate resins as the carrier materials, indicated the possibility for retarding, sustaining, prolonging, or even controlling the drug release [20,21]. Other lipid carriers, such as cholesterol, tristearin, β-sitosterol, and carnauba wax, have also been suggested as possible matrix materials for drug-release applications [22,23].

In order to provide the native EM with protection from sunlight photolysis, two polymers (one water-soluble and one no-water-soluble) are used as its carrier materials by preparing solid dispersions. Protection from photodegradation would be succeeded by the sustained release of EM, i.e., EM would release in small amounts and the other amounts dispersed in the polymeric matrices would be protected. In advance, it was expected that PVP-K30, as water-soluble polymer, would improve the solubility and dissolution rate of EM, leading to an increased concentration of the molecular EM in the aqueous solution, something that may enhance the insecticidal activity of EM against its target pests as *Plutella xylostella*. Moreover, polyacrylic resin (PR) III, as a water-insoluble polymer, was expected to improve the long-term insecticidal activity of EM under practical agricultural field application conditions.

## 2. Experimental

### 2.1. Materials

All experiments were conducted on the Fujian Agriculture and Forestry University (FAFU) campus (34°480N, 113°180E), Fuzhou, Fujian, China. A field population of *P. xylostella* was collected from the commercial crucifer fields at Shangjie (Sj), which is 20 km away from FAFU. No specific permits were required for the collection of *P. xylostella*, and no endangered or protected species were involved. The experimental protocol used for the present study complied with the regulatory recommendation and we obtained the necessary approval to carry out the study. Emamectin benzoate (EM) (technical grade 95.8% purity) was purchased from Jiamusi Xingyu Biotechnique Development Co., Ltd., Heilongjiang, China. Polyvinyl pyrrolidone (PVP) (technical grade, K30) was purchased from Sinopharm Chemical Reagent Limited Corp., Co., Ltd., Beijing, China. Polyacrylic resin (PR) (technical grade, III) was purchased from Shanghai Lichen Biotechnique Co., Ltd., China. Methanol used for the study was of high-performance liquid chromatography (HPLC) grade. Spectrographic grade KBr used for infrared spectroscopy sample preparation. The supplier of methanol and KBr was China National Pharmaceutical Group Corporation. All other reagents were of analytical grade.

### 2.2. Preparation of Solid Dispersions

#### 2.2.1. Preparation of EM-PVP-K30 Solid Dispersions

A solution of EM in methanol (12.5% *w*/*v*) and a solution of PVP-K30 in methanol (25% *w*/*v*) were mixed at 600 rpm in a beaker for 60 min at a certain feed ratio of EM:PVP-K30 (m/m). The mixture was transferred to a mortar (diameter: 16 cm), and the methanol was volatilized by keeping the mortar in a dark ventilated place for 48 h. The solid dispersions formed gradually upon manual grinding of the mixture. After drying the solid dispersions at 50 °C for 24 h, the product was crushed using a mill and was passed through an 80-mesh sieve. The resulting EM-PVP-K30 solid dispersions were stored in a valve bag, which was placed in a glass dryer at ambient temperature, away from direct sunlight. Blank PVP-K30 solid dispersions were prepared using the same procedure without the addition of EM.

#### 2.2.2. Preparation of EM-PRIII Solid Dispersions

A 10% *w*/*v* methanol solution of PRIII was prepared in a mortar (diameter: 16 cm) by grinding, to which was added a 12.5% *w*/*v* solution of EM in methanol. The mixed solution was ground constantly for 60 min at room temperature and was then placed in a dark ventilated place for 48 h to volatilize the methanol. After grinding manually and drying the solid dispersions at 50 °C for 24 h, the product was crushed with a mill and was passed through an 80-mesh sieve. The EM-PR III solid dispersions were sealed and kept in a glass dryer at room temperature in the dark. Blank PRIII solid dispersions were prepared using the same method without the addition of EM.

The preparation process of 10% *w*/*v* methanol solution of PRIII was as follows. The PRIII was crushed into powder by a high-speed grinder, and then grinded to a clear solution with methanol in a mortar. This ensured that most of the PRIII was dissolved in methanol. Moreover, all EM and blank -PRIII solid dispersion samples were prepared with this kind of PRIII methanol solution. Filtration was not conducted in order to ensure the consistency of the experimental conditions. The PRIII that was possibly undissolved finally participated in solid dispersion formulations since they were conducted under manual grinding and evaporated the solvent.

### 2.3. Effects of Different Factors on the Characteristics of Solid Dispersions

A single-factor exploration method was used to study the influence of different factors, including feed ratio, solvent, rotational speed, and mixing time on the pesticide loading and the entrapment rate of solid dispersions. At least three replicates were performed for each experiment.

#### 2.3.1. Feed Ratio

Different feed ratios of the EM:PVP-K30 and EM:PRIII (m/m) solutions (1:1, 1:2, 1:4, 1:10, 1:50, and 1:100) were evaluated during the preparation of solid dispersions in order to study their effect on the entrapment rate.

#### 2.3.2. Solvent

The solution of EM (0.21 g) was prepared in different single common organic solvents (50 mL), such as methanol, ethanol, acetone, acetonitrile, ethyl acetate, or dichloromethane at room temperature (20 ± 2 °C). The dissolution phenomenon and time required for the complete dissolution were observed and recorded. After completing the solvent screening for optimization, the optimal solvents were used in a 1:1 feed ratio for the preparation of solid dispersions to investigate the influence of different solvents on the characteristics of the prepared solid dispersions. Thus, the solutions were PVP-K30 (1.0 g) and PRIII (1.0 g).

#### 2.3.3. Rotational Speed and Mixing Time

The EM-PVP-K30 solid dispersions were prepared by using different rotational speeds (400, 600, and 800 rpm), with a feed ratio of 1:1 and a 60 min mixing time. Additionally, different mixing times (20, 40, 60, 80, and 100 min) were also employed for the preparation of EM-PVP-K30 solid dispersion with the 1:1 feed ratio at 600 rpm. The assay measurements for the pesticide loading and entrapment rates of solid dispersions were determined with more than three replicates for each assay.

### 2.4. Characteristics of Solid Dispersions

#### 2.4.1. Entrapment Rate and Pesticide Loading

The entrapment rate was defined as the ratio of actual-to-theoretical pesticide content in the dispersion. The pesticide loading was defined as the actual content of EM in solid dispersions. The EM-PVP-K30 solid dispersions containing about 0.02 g of EM were dissolved using 50 mL of methanol, and the EM-PRIII solid dispersions were dissolved in methanol under water bath heating at 50 °C. After sonication for 60 min at 25 °C in the dark, the methanol solution was further diluted to 100 mL. EM concentration in the methanol solution was determined by HPLC with an Amemyst C18-H column at 25 °C. The mobile phase was composed of a methanol/water/triethylamine solution (10 %, 92:7:1), and a flow rate of 1.0 mL/min was used. The detection wavelength was 244 nm, and the injection quantity was 20 μL. From the determined concentration, the entrapment rate and content of EM in the prepared solid dispersions were calculated. Additionally, more than three replicates were performed for each assay.

#### 2.4.2. X-ray Powder Diffraction (XRD)

The XRD analysis (Bruker, Co., Ltd., Germany) of the four samples (>100 mg each), EM, EM-PVP-K30 (or EM-PRIII) solid dispersions prepared under varied feed ratios, a physical mixture of EM and PVP-K30 (or PRIII), and PVP-K30 (or PRIII), were carried out. The samples were ground further and were passed through an 80-mesh sieve and were dried before the measurements were conducted. A 2θ angle range of 0–50° and a 6 °/min scanning speed were used along with a 0.02 s time of each scanning step.

#### 2.4.3. Differential Scanning Calorimetry (DSC) and Thermogravimetric (TG) Analysis

Ten milligrams of EM, EM-PVP-K30 (or EM-PRIII) solid dispersions, a physical mixture of EM and PVP-K30 (or PRIII) (m/m = 1:10), and PVP-K30 (or PRIII), were used for the DSC and TG analysis. Thermal analyses were performed from 25 to 800 °C using a DSC instrument (Netzsce, Co., Ltd., Germany) with a heating rate of 10 °C/min under an argon atmosphere.

#### 2.4.4. Ultraviolet (UV) Spectrometry

The samples of EM, PVP-K30 (or PRIII), EM-PVP-K30 (or EM-PRIII) solid dispersions, and a physical mixture of EM and PVP-K30 (or PRIII) were dissolved in methanol and the solutions were analyzed by UV spectrometry. A 1:10 feed ratio was maintained for the EM-PVP-K30 (or EM-PRIII) solid dispersions and the physical mixture of EM and PVP-K30 (or PRIII). Methanolic solutions of each sample containing EM were prepared at a concentration of 0.02 mg/mL. Methanolic solutions of PVP-K30 (or PRIII) were prepared at a concentration of 0.20 mg/mL. UV Spectrometry of these solutions was carried out in the 190–300 nm wavelength region, using pure methanol as the blank.

#### 2.4.5. Infrared (IR) Spectroscopy

The sample preparation for IR spectrometry was carried out by using 2 mg of the four dry samples of EM, EM-PVP-K30 (or EM-PRIII) solid dispersions, a physical mixture of EM and PVP-K30 (or PRIII), and PVP-K30 (or PRIII) for the preparation of the potassium bromide pellet. Each sample was ground with 400 milligrams of dry potassium bromide in an agate mortar. The mixed powder was added to the mold and was packed into a solid sample frame and pressed into a pellet, which was used for analysis by IR spectrometry (Tuopu instruments, Co., Ltd., Tianjin, China). The measurements were carried out in the transmittance mode (0–100) by continuous scanning with a fast scanning speed and a normal slit width and response time, in the 4000–400 cm^−1^ wavenumber region. A scanning time of 3 was used, and the blank control was a pure potassium bromide pellet.

#### 2.4.6. Morphology of Solid Dispersions

Dry solid dispersion samples were sputter-coated with gold by using a JFC-1200 Sputter Coater 9 (Japan Electron), and the morphology of the solid dispersions were analyzed by scanning electron microscopy (SEM) (JSM-5310LV, Japan Electron).

### 2.5. Aqueous Solubility of EM-PVP-K30 Solid Dispersions

The samples of EM, EM-PVP-K30 solid dispersions prepared by using a varied feed ratio, and a physical mixture of EM and PVP-K30 (m/m = 1:10), whose quantities were greater than their maximum solubility in water, were poured into Erlenmeyer flasks containing 100 mL of ultra-pure water. The Erlenmeyer flasks were wrapped and sealed with tin foil and were placed in a constant temperature oscillation incubator at 20 °C at an oscillation rate of 100 rpm for 3 h. The aqueous solubility of samples was measured after the suspensions were allowed to stand for 20 min. A 5 mL aliquot was taken and was centrifuged at 20 °C at 8000 rpm for 10 min, and 1 mL of the collected supernatant was diluted with ultra-pure water in a 10 mL brown glass volumetric flask. EM concentration of the three samples in their saturated solutions was determined by HPLC. The residual solutions were kept in the constant temperature oscillation incubator that was used previously, and their EM concentrations were measured after every 60 min until the difference between two consecutive aqueous solubility measurements was less than 3%. At least three replicates were performed for each assay. The aqueous solubility of the three samples was ten times more than EM concentration of their saturated solutions.

### 2.6. Sustained-release of EM-PRIII Solid Dispersions

#### 2.6.1. Sustained-Release in pH 7.0 Phosphate Buffer Solution

Forty-five copies of the EM technical sample and the samples of the solid dispersions prepared using different EM-PRIII (m/m) feed ratios (1:1, 1:4, 1:10, 1:50, and 1:100) were each weighed and placed in separate 100 mL Erlenmeyer flasks. All samples contained 0.02 g of EM. Fifty mL of pH 7.0 phosphate buffer solutions were then added to the Erlenmeyer flasks. These samples were sealed and kept in a dry and dark place, and the suspensions were filtered by suction after 1, 2, 4, 8, 12, 18, 24, and 36 h, and 2, 4, 6, 8, 10, 15, and 20 d. The filtered solids from each sample were collected and dissolved in methanol. The release rates of the EM-PRIII solid dispersions and EM technical samples in buffer solutions were determined by HPLC after the samples were kept under a dry and dark condition for different times. At least three replicates were performed for each assay.

#### 2.6.2. Sustained-Release in pH 5.8 and 7.8 Phosphate Buffer Solutions

The release rates of the EM-PRIII solid dispersions (feed ratio = 1:10) and EM technical samples in the pH 5.8 and 7.8 phosphate buffer solutions were determined by the method described in Section 2.6.1.

### 2.7. Photolysis Stability

The samples of EM, EM-PVP-K30, and EM-PRIII solid dispersions were dissolved in water (concentration: 0.3 mg/mL). The aqueous solutions (1 mL) were added into respective dry Petri dishes (diameter: 16 cm) with a pipette (Biohit, Co., Ltd., Finland). The solutions were spread evenly in the dishes by slight oscillation and were then placed on the surface of a three-functional UV analyzer (wavelength: 254 nm, power: 4 W, filter size: 200 mm * 50 mm). The distance between the bottom of the dishes and the filter plate was 144 mm. The degradation rates of EM in the three samples were determined by HPLC after 0, 1, 2, 4, 6, 8, 10, and 12 h. The insecticide degradation rate at a particular time was calculated as the ratio of the concentration of EM at that time vs. zero time. A kinetic equation was established, and more than three replicates were performed for each assay [24].

### 2.8. Wettable powder Preparation of 5 % Solid Dispersions (m/m)

A proper quantity of EM-PVP-K30 solid dispersion, prepared with a 1:10 feed ratio of EM:PVP-K30, 55 g, was composed of the addition of 5 g of anhydrous sodium sulfate, 2 g sodium dodecyl benzene sulfonate, and 38 g of diatomite. Crushing, mixing, and drying of the mixture afforded the wettable powder a 5% EM-PVP-K30 solid dispersion. The wettable powder of 5% EM-PRIII solid dispersion was prepared similarly, using 5 g of sodium dodecyl benzene sulfonate and 35 g of diatomite.

### 2.9. LC_50_ Bioassay

Using a leaf dipping method [25,26,27], the toxicity of the wettable powder suspension of EM-PVP-K30 (or EM-PRIII) solid dispersion was determined at 25 °C and was compared with that of the third-instar larvae of *P. xylostella*. A photoperiod of 16:8 (light:dark) was used for this study in the laboratory. On the basis of previous tests, the concentrations at which the corrected death rate ranged from 10% to 90% were selected as the treatment concentrations. Discs of cabbage (*Brassica oleracea* L.) leaves, 5 cm in diameter, were dipped in these pre-prepared suspensions for 10 s and were then taken out and air-dried naturally. Subsequently, the leaves were placed into Petri dishes containing wet filter papers, and new third-instar larvae of *P. xylostella* were placed in the Petri dishes (10 larvae per replication). Five biological replicates and controls were used for each solid dispersion concentration. Mortalities of the *P. xylostella* larvae were recorded after 24 h. The larvae were considered dead if no autonomous response was observed when they were touched with a writing brush. Zero mortality was observed in the suspension without the insecticide (control). The number of test deaths was recorded, and the adjusted mortality rate was calculated for each treatment. If the mortality rate of the control group was less than 5%, no adjustment was performed. If the mortality rate of the control group was between 5% and 20%, the adjusted mortality rate was calculated. If the mortality rate of the control group was more than 20%, the test was repeated.

### 2.10. Sustained-release Effect of the Wettable Powder of 5 % EM-PRIII Solid Dispersions

The wettable powder suspension of EM-PR III solid dispersion, whose concentration was 0.146 mg/mL (approximate LC_90_ value) was sprayed on the cabbage (*Brassica oleracea* L.) plants with a hand-held compression sprayer. Leaves were taken after 0, 5, 10, 15, and 20 d, and were placed into Petri dishes containing wet filter papers. Fresh third-instar larvae of *P. xylostella* were placed in the Petri dishes (10 larvae per replication). Four biological replicates were obtained, and the mortalities of the *P. xylostella* larvae were recorded after 48 h. If the larvae had no autonomous response when they were touched with a writing brush, they were considered dead. The commercial cabbage plants, which were treated by spraying with 1.5 % emulsifiable EM concentrate (*w*/*v*), were used as contrasting counterparts. In addition, cabbage plants sprayed with distilled water were used as a control.

## 3. Results and Discussion

### 3.1. Effects of Different Factors on the Characteristics of Solid Dispersions

#### 3.1.1. Feed Ratio

**Figure 1:**
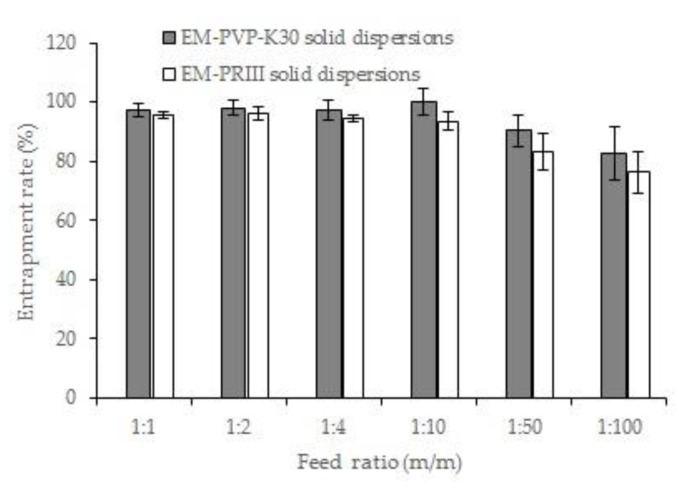
Effects of different feed ratios on the entrapment rate of solid dispersions.

Studies on the influence of feed ratio on the entrapment rate revealed that entrapment rates greater than 97% (EM-PVP-K30 solid dispersions) and 93% (EM-PRIII solid dispersions) were achieved when the feed ratio of EM:PVP-K30 and EM:PRIII (m/m) was increased from 1:1 to 1:10. In addition, the entrapment rate of solid dispersions indicated a tendency to decrease with an increase in the ratio of PVP-K30 (or PR III) in solid dispersions from 1:10 to 1:100. In particular, when the feed ratio was 1:50 and 1:100, the entrapment rate of the EM-PVP-K30 solid dispersions was 90.23% ± 5.13%, and 82.81% ± 8.97%, respectively, and the EM-PRIII solid dispersions were 83.20% ± 6.00% and 76.21% ± 6.97%, respectively. Furthermore, the dispersion degree of the entrapment rate increased with the decreasing ratio of EM in solid dispersions (Figure 1). These results indicated that EM and carrier materials could not be mixed well in solid dispersions when the feed ratio was too large. On the basis of these results, we concluded that the feed ratio should be between 1:1 and 1:50.

#### 3.1.2. Solvent

**Figure 2:**
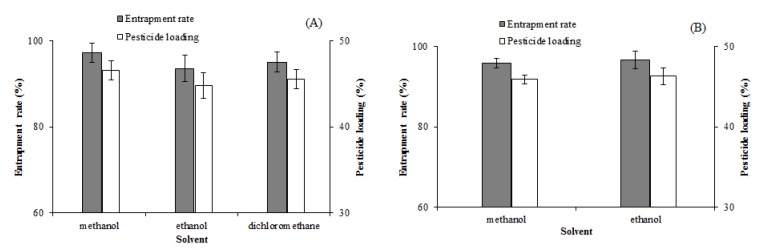
Effects of different solvents on the entrapment rate and pesticide loading of solid dispersions. (**A**): EM-PVP-K30 solid dispersions. (**B**): EM-PRIII solid dispersions.

The selection of a suitable solvent for dissolving EM and the carrier materials was necessary when the solid dispersions were prepared by the solvent method. The solubility of PVP-K30 and PRIII in common organic solvents, which can dissolve EM, such as methanol, ethanol, acetone, acetonitrile, ethyl acetate, and dichloromethane, were evaluated. PVP-K30 is soluble in methanol, ethanol, and dichloromethane, and PRIII is soluble in methanol and ethanol. Having assessed the solubilities, the EM-PVP-K30 solid dispersions and the EM-PRIII solid dispersions were prepared using the optimal solvents. The entrapment rate and pesticide loading of EM-PVP-K30 solid dispersions prepared using methanol, ethanol, and dichloromethane were higher. However, they did not significantly differ from each other (Figure 2A). The EM-PRIII solid dispersions prepared using methanol and ethanol indicated a similar trend (Figure 2B). However, methanol was a better solvent for dissolving both EM and PVP-K30 than the other two solvents (Table 1). When compared to ethanol, whose saturated vapor pressure is 5.87 kPa (at 20 °C), methanol has a higher saturated vapor pressure (12.97 kPa at 20 °C) [28], and therefore methanol was the suitable solvent of preparation of EM-PRIII solid dispersions, by evaporating the solvent in a facile manner. Therefore, methanol was chosen as the solvent for preparing both EM-PVP-K30 and EM-PRIII solid dispersions.

#### 3.1.3. Rotational Speed and Mixing Time

**Figure 3:**
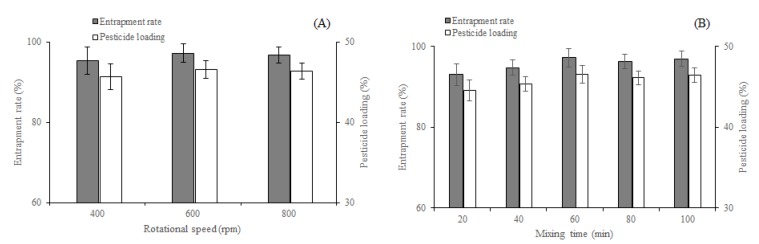
Effects of (**A**) rotational speed and (**B**) mixing time on entrapment rate and pesticide loading of EM-PVP-K30 solid dispersions.

The mixing degree of EM and PVP-K30 in the solvent would have influenced the entrapment rate and pesticide loading of solid dispersions. Therefore, the rotational speed and the mixing time might be two important factors that could influence the preparation procedure of the dispersions. To study the impact of these factors, we conducted several experiments, and the results indicated an increase in the entrapment rate and pesticide loading with an increase in the rotational speed from 400 r/min to 600 r/min. While the differences were not significant, the entrapment rate and pesticide loading increased from 45.68% ± 1.62% and 95.36% ± 3.39% to the highest rate of 46.56% ± 1.09% and 97.21% ± 2.27%, respectively (Figure 3A). Therefore, the rotational speed of 600 r/min was chosen as the optimal condition. However, the pesticide loading and entrapment rate increased with an increase in the mixing time from 20 to 60 min, but no significant differences could be found when the mixing time was increased from 60 to 100 min (Duncan’s tests [29], *p* ≤ 0.05) (Figure 3B). Therefore, a mixing time of 60 min was chosen as the optimal time range.

### 3.2. Solid Dispersions Characteristics

#### 3.2.1. XRD

**Figure 4:**
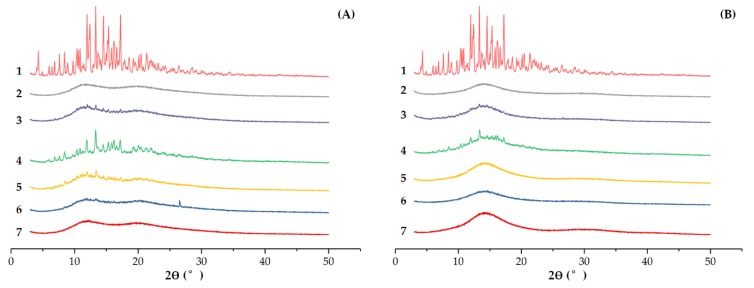
XRD of (**A**) EM-PVP-K30 solid dispersions. (**B**) EM-PRIII solid dispersions. (**A**): (1) EM, (2) PVP-K30, (3) physical mixture of EM and PVP-K30, (4) 1:1 feed ratio EM-PVP-K30 solid dispersions, (5) 1:4 feed ratio EM-PVP-K30 solid dispersions, (6) 1:10 feed ratio EM-PVP-K30 solid dispersions, (7) 1:50 feed ratio EM-PVP-K30 solid dispersions; (**B**): (1) EM, (2) PRIII, (3) physical mixture of EM and PRIII, (4) 1:1 feed ratio EM-PRIII solid dispersions, (5) 1:4 feed ratio EM-PRIII solid dispersions, (6) 1:10 feed ratio EM-PRIII solid dispersions, and (7) 1:50 feed ratio EM-PRIII solid dispersions.

The XRD spectra of EM, PVP-K30, PRIII, physical mixture, and solid dispersions were obtained (Figure 4). EM was obviously in its crystalline form in all samples. The inability to observe significantly diffraction peaks in 1:50 feed ratios of solid dispersions might be due to the sensitivity of the technique used.

#### 3.2.2. DSC and TG Analysis

**Figure 5:**
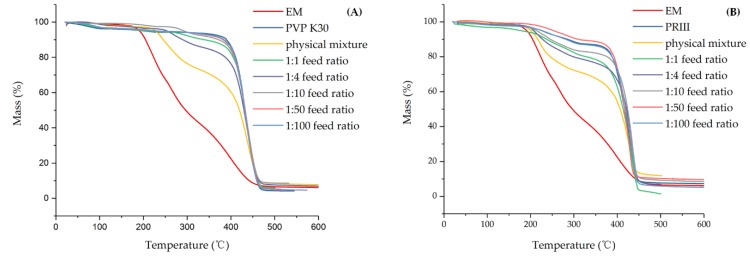
TG of (**A**) EM, PVP K30, physical mixture, and EM-PVP-K30 solid dispersions. (**B**) EM, PRIII, physical mixture, and EM-PRIII solid dispersions.

**Figure 6:**
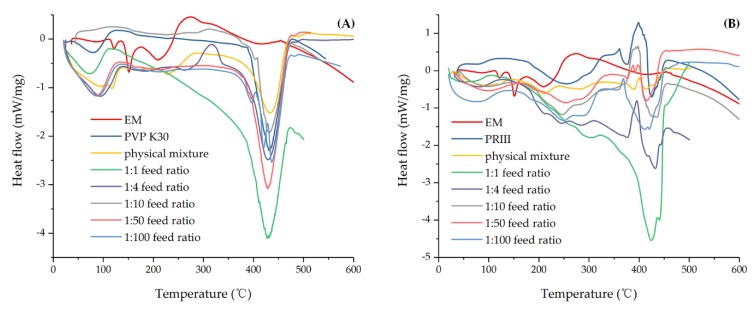
DSC of (**A**) EM, PVP K30, physical mixture, and EM-PVP-K30 solid dispersions. (**B**) EM, PRIII, physical mixture, and EM-PRIII solid dispersions.

The TG analysis results are shown in Figure 5; the major weight loss of EM began at around 200 °C and solid dispersions rose to nearly 400 °C. According to the TG curve, only 22.48% of EM remained at 400 °C, PVP K-30 and PRIII were more stable than EM, and the residual weights were 86.37% and 70.78%, respectively. Meanwhile, the physical mixture of EM and PVP-K30 (m/m=1:10), solid dispersions with feed ratios of 1:1, 1:4, 1:10, 1:50, and 1:100, at the same temperature, were 60.23%, 82.22%, 76.33%, 84.05%, 85.35%, and 86.17%, respectively. Additionally, 54.23%, 62.80%, 66.65%, 72.52%, 73.59%, and 72.61% of physical mixture of EM and PRIII (m/m = 1:10), solid dispersions with feed ratios of 1:1, 1:4, 1:10, 1:50, and 1:100, remained at 400 °C. These might suggest a stabilization effect of EM by forming PVP K-30 and PRIII solid dispersions. In Figure 6, the DSC diagram of EM exhibited a sharp endothermic peak around 150 °C, indicating the melting point of EM, followed by an exothermic peak at about 200 °C, which might be attributable to the decomposition of EM and was confirmed by TG analysis. During scanning of the PVP-K30, a broad endotherm ranging from around 40 to 120 °C was observed, indicating the loss of water due to the extremely hygroscopic nature of PVP polymers. The sharp endothermic peak that began around 400 °C might have been at the beginning of the decomposition of PVP-K30, which was also verified by TG analysis. Meanwhile, the PRIII can be observed as a slight endothermic curve, in the interval between 180 and 320 °C, where about 150 °C represented the melting point of PRIII. From the TG and DSC diagrams, the degradation process of PRIII began at about 400 °C. The physical mixture of EM and PVP-K30 (or PRIII) (m/m = 1:10) showed the endothermic and decomposition peak of EM and the broad endothermic peak belonging to PVP (or PRIII). As for the EM-PVP-K30 solid dispersions, the endothermic peak representing the melting point of EM was rarely observed, and the exothermic peak was ascribed to the decomposition of EM that shifted to the high temperature in all samples. In the case of EM-PRIII solid dispersions, no endothermic peak was observed around 120 °C and 150 °C. The shifting of the melting point to the higher temperature around 250 °C may have been a reason for the interaction of EM with PRIII. These results might show the evidence of forming solid dispersion between EM and the PVP-K30 or PRIII carrier materials.

#### 3.2.3. UV Spectrometry

**Figure 7:**
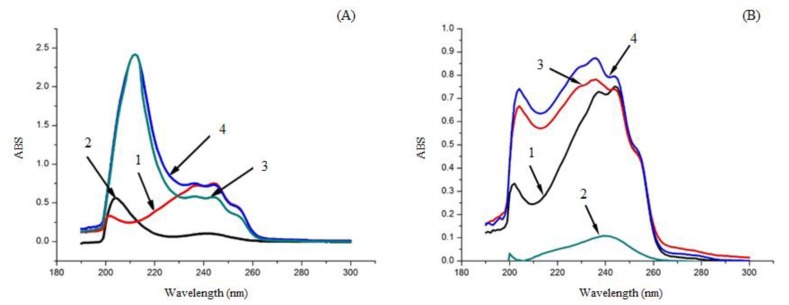
UV Spectrometry of different samples. (**A**): 1) EM, 2) PVP-K30, 3) EM-PVP-K30 solid dispersions, 4) physical mixture of EM and PVP-K30; (**B**): 1) EM, 2) PRIII, 3) EM-PRIII solid dispersions, and 4) physical mixture of EM and PRIII.

The UV absorption peak shapes of EM, solid dispersions, and the physical mixtures were similar in the 200–260 nm wavelength region. However, analysis of these three samples with identical concentrations in methanol indicated detectably lower absorption of the solid dispersions than that of EM or the physical mixture (Figure 7). Concerning physical mixtures and solid dispersions with PVP, the spectra in UV were identical, meaning that homogenous fixing had occurred. According to Figure 4B, the spectra of EM-PRIII solid dispersions was slight and the peaks were not well separated, showing that bond formation occurred. All the results implied that EM and both the other carriers, PVP-K30 and PRIII, were not sufficiently mixed physically during their preparation, and bonded together into a new form.

#### 3.2.4. IR Spectrometry

**Figure 8:**
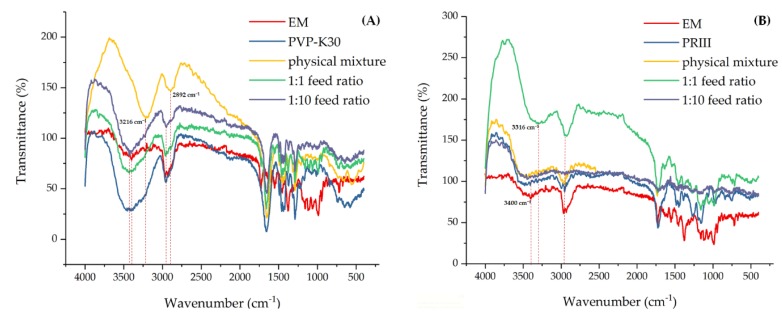
IR Spectra of (**A**) EM, PVP K30, physical mixture, and EM-PVP-K30 solid dispersions. (**B**) EM, PRIII, physical mixture, and EM-PRIII solid dispersions.

The IR spectra of EM, PVP-K30, and PRIII are presented in Figure 8 and the wavenumber data from these spectra are summarized in Table 2. The IR spectra of the EM-PVP-K30 and EM-PRIII solid dispersions indicated the presence of characteristic groups. Further, the EM-PVP-K30 solid dispersions, prepared with 1:1 and 1:10 feed ratios of EM:PVP-K30 (m/m)), showed similar IR fingerprints. However, some change occurred in the feature region of the IR spectra of solid dispersions prepared in 1:1 and 1:10 feed ratios. In the feature region, the 3400 cm^−1^ –OH stretching vibration peak of EM and 3416 cm^−1^ -NH stretching vibration of PVP-K30 shifted to a higher wavenumber band. That is, the peak shifted to 3428 cm^−1^ and 3432 cm^−1^ in the 1:1 and 1:10 feed ratios (EM:PVP-K30) solid dispersions, respectively. In contrast to the physical mixture, whose O-H or N-H stretching vibration and C=O stretching vibration peak was 3216 cm^−1^ and 2892 cm^−1^, obvious ‘blue shift’ could also be observed for the solid dispersions. It seemed that an intermolecular hydrogen bond between O–H of EM and N–H of PVP-K30 formed. Due to the amount of PRIII being higher in both the physical mixture and the EM-PRIII solid dispersions whose feed ratios of EM:PRIII (m/m) were 1:10, the 3400 cm^−1^ O–H stretching vibration peak of EM were hardly discernible in the IR spectra. However, obvious ‘red shift’ could be observed for the solid dispersions with 1:1 feed ratios (EM:PRIII), that is, the peak was 3316 cm^−1^. In addition, when the solid dispersions were prepared in 1:10 feed ratios of EM:PRIII (m/m), C–H stretching vibration (saturated bond) peaks, which were 3000–2900 cm^−1^ in the feature region, were hardly observed. It also implied that a hydrogen bond formed between EM and PRIII at a molecular level. However, the XRD spectra showed that EM was obviously in its crystalline form in both EM-PVP-K30 and EM-PRIII solid dispersions.

#### 3.2.5. Morphology of Solid Dispersions

**Figure 9:**
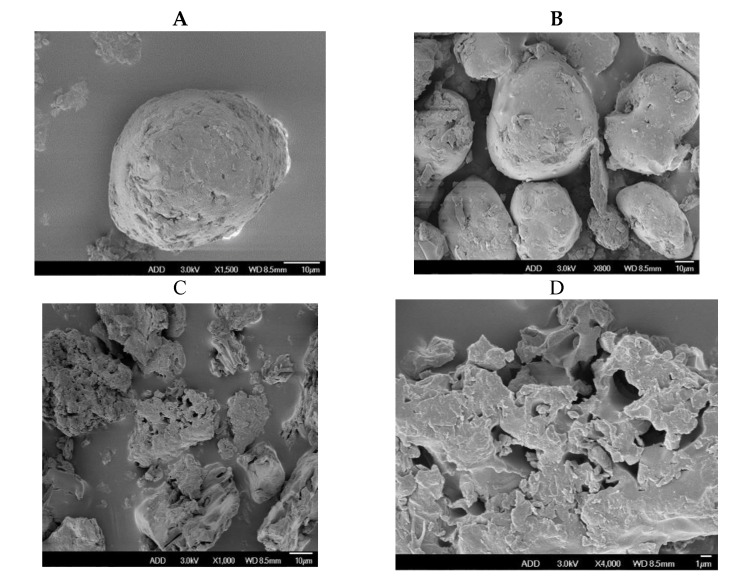
Scanning electron micrographs of (**A** + **B**) EM-PVP-K30 solid dispersions and (C + **D**) EM-PRIII solid dispersions.

The EM-PVP-K30 solid dispersions looked round, irregular, and potato-like with a ridged surface. Compared with the EM-PVP-K30 solid dispersions, the EM-PRIII solid dispersions were irregular solids, with a microporous structured-surface (Figure 9). Based on these results, it can be speculated that the EM embedded in the PVP-K30 carrier material was released from the micropores present in EM-PRIII solid dispersions.

### 3.3. Aqueous Solubility of EM-PVP-K30 Solid Dispersions

One of the key aims of the study was to increase the solubility of EM by preparing its EM-PVP-K30 solid dispersions. The aqueous solubility of EM was determined to be 23.36 × 10^−3^ mg/mL from our studies described in Section 2.5, which is in good agreement with the literature value of 24.00 × 10^−3^ mg/mL [3]. It is noteworthy that the aqueous solubility of EM indicated a significant increase (Table 3) in its EM-PVP-K30 solid dispersion form. Upon increasing the PVP-K30 content in EM-PVP-K30 solid dispersions, their aqueous solubility increased to a maximum value when the feed ratio of EM:PVP-K30 was 1:10, which is 37.5 times the aqueous solubility of EM. The aqueous solubility began to decrease with a further increase in the ratio of EM:PVP-K30. In contrast, the aqueous solubility of the physical mixture of EM and PVP-K30 prepared in a 1:10 ratio was only 3.3 times more than that of EM. Therefore, EM and PVP-K30 did not exist as simple physical mixtures in the solid dispersions, but existed in a molecular or amorphous state, which improved their solubility in water.

### 3.4. Sustained-release of EM-PRIII Solid Dispersions

#### 3.4.1. Sustained-release in pH 7.0 Phosphate Buffer Solution

**Figure 10:**
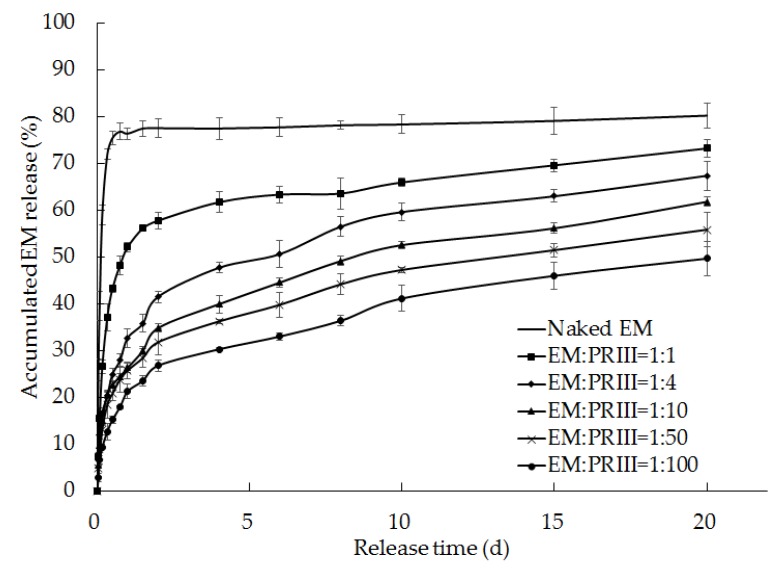
Release rates of EM from EM-PRIII solid dispersions prepared using varied feed ratios.

With an increase in the PRIII content, the release rate of EM from EM-PRIII solid dispersions slowed down. On the first day, about 71.91% ± 1.13% of EM technical was released, and the release rates of solid dispersions with feed ratios of 1:1, 1:4, 1:10, 1:50, and 1:100 were 37.07% ± 2.88%, 20.35% ± 0.68%, 20.64% ± 0.85%, 18.36% ± 2.97%, 12.61% ± 1.81%, respectively. Additionally, on the fourth day, the release rates of EM technical and these solid dispersions were increased to 77.49% ± 2.32%, 61.71% ± 2.28%, 47.70% ± 1.18%, 39.93% ± 1.88%, 36.24% ± 0.48%, 30.28% ± 0.42%, respectively. Over 20 d, 80.23% ± 2.60% of EM technical was released, whereas the EM enclosed in the solid dispersions prepared with a 1:100 feed ratio of EM:PRIII was released at 49.66% ± 3.66% (Figure 10). Compared with the EM technical, whose half-life was only 0.12 d, the half-life of EM was extended from 0.67 to 16.76 d, when the feed ratio of EM:PRIII in the solid dispersions was increased from 1:1 to 1:100 (Table 4). Consequently, the solid dispersions using PRIII as the carrier materials afforded a significant advantage for prolonging the insecticide release.

#### 3.4.2. Sustained-release in pH 5.8 and 7.8 Phosphate Buffer Solutions

**Figure 11:**
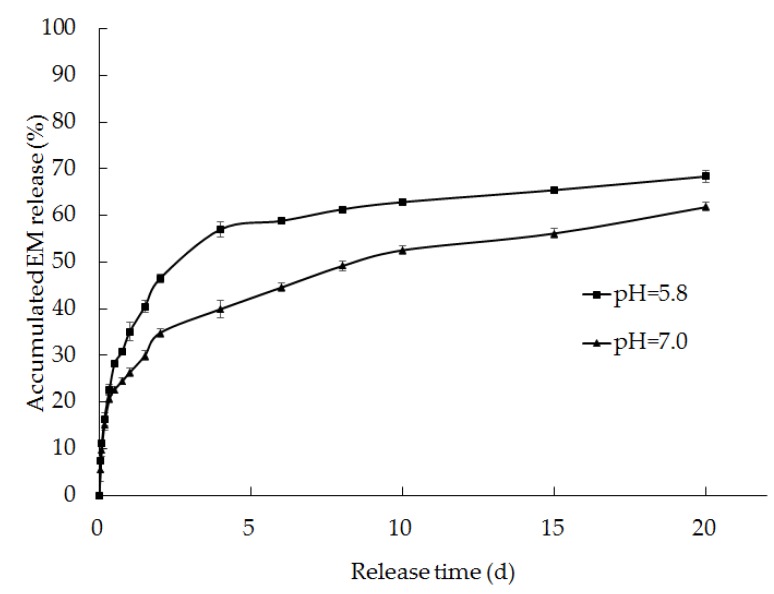
Release rates of EM from the solid dispersions prepared using a 1:10 feed ratio of EM-PRIII in phosphate buffer solutions of pH 5.8 and 7.0.

The release profiles of EM in the solid dispersions, prepared with the 1:1 feed ratio of EM-PRIII, were determined in pH 5.8 and 7.8 phosphate buffer solutions. In the pH 7.8 phosphate buffer solution, the decomposition rate of EM was high, and there was little solid residue left in the Erlenmeyer flask after 1 d. However, the release rate of EM in the pH 5.8 phosphate buffer solution was faster than that in the pH 7.0 phosphate buffer solution (Figure 11). The kinetic equation for the EM release rate in the pH 5.8 phosphate buffer solution can be described by Q = 61.886 (1 − e^-0.914t^). The correlation coefficient for this equation is 0.95, and it can be classified as a first-order equation. In contrast, the kinetic equation for the release rate in the pH 7.0 phosphate buffer solution was classified as a Higuchi equation.

### 3.5. Photolysis Stability

Upon exposure to UV light, the insecticide in the EM-PVP-K30 and EM-PRIII solid dispersions degraded significantly less in comparison to the unmodified EM. Importantly, the half-life of EM increased with an increase in the content of carriers, such as PVP-K30 or PRIII in the solid dispersions (Table 5 and Table 6). These results demonstrated that the solid dispersion of EM afforded a significant advantage for preserving the efficacy of the insecticide by reducing the adverse effect of photolysis by both UV and sunlight.

### 3.6. LC_50_ Bioassay

Based on the mortality of the pests at 24 h, the toxicity regression equations and lethal concentrations 50 (LC_50_) were calculated. The LC_50_ of EM technical, EM-PVP-K3, and the wettable powder of EM-PRIII solid dispersion were 23.0 μg/mL, 12.3 μg/mL, and 31.3 μg/mL, respectively (Table 7). These results demonstrated that the wettable powder formed by EM-PVP-K30 solid dispersion could enhance the insecticidal activity of EM technical. In contrast, there were no significant differences between the insecticidal activity of wettable powder of EM-PRIII solid dispersion and that of EM technical.

### 3.7. Sustained-release Effect of the Wettable Powder of 5% EM-PRIII Solid Dispersion

The wettable powder of 5% EM-PRIII solid dispersions and 1.5% EM emulsifiable concentrate were sprayed on the cabbage plants that were grown in the laboratory and were kept in the natural environment for different durations, and the degradation of EM by photolysis and hydrolysis was examined. At 0 d (immediately after the preparation), no degradation was observed, and the toxicity of the wettable powder of EM-PRIII solid dispersion was 83.19%, which was lower than that of the EM emulsifiable concentrate (94.69%). After 5 d, there was no significant difference in insecticidal activity between the two preparations. However, after 10 d, the toxicity of EM emulsifiable concentrate declined significantly and practically disappeared on the final day of the test. In contrast, the wettable powder of EM-PRIII solid dispersion maintained a higher insecticidal activity than its counterpart for more than 20 d (Table 8).

## 4. Conclusions

In this study, different formulations of the biosynthetic derivative of abamectin and emamectin benzoate were evaluated to improve its solubility. Fast-release and sustained-release solid dispersion formulations of EM were prepared using PVP-K30 and PRIII, respectively, and employed a solvent method, which is traditionally used for the solid dispersion preparation of high temperature-sensitive or volatile drugs and materials. Both PVP-K30 and PRIII were used as carrier materials. PVP-K30 has good water-solubility and is widely used as a carrier for the preparation of solid dispersions of poorly water-soluble drugs for improving their solubility and dissolution rate [30,31,32]. PRIII is an enteric-soluble and slow-release material, which is mainly used as a capsule coating agent, and an enteric coating and sustained-release preparation material [33].

(1) Based on the observed entrapment rates and pesticide loadings, the processes for preparing the solid dispersions were optimized.

(2) To confirm the formation of solid dispersions and to investigate the relationships that govern the fast or sustained-release of EM and the carriers, XRD, DSC, UV, IR, and scanning electron microscopy were employed to characterize the solid dispersions [34,35], and the aqueous solubility of EM-PVP-K30 solid dispersions. The XRD spectra showed that EM was obviously in its crystalline form in both EM-PVP-K30 and EM-PRIII solid dispersions. The results of DSC, UV, and IR implied that these two types of solid dispersions were formed by intermolecular hydrogen bonds between EM and its carrier materials. This could be the underlying reason for the improved solubility and dissolution rate of EM by solid dispersion in PVP-K30. The aqueous solubility reached the maximum when the feed ratio of EM:PVP-K30 was 1:10, which might be related to the completely different DSC curve. Notably, the half-life of EM could be extended from 0.67 d to 16.76 d by forming EM-PRIII solid dispersions by increasing the feed ratio of EM:PRIII from 1:1 to 1:100 (the half-life of EM technical was 0.12 d). The effect of pH on the enteric solubility of PRIII was studied, and the order of the EM release rate from EM-PRIII solid dispersions in different phosphate buffer solutions was found to be pH 7.8 > pH 5.8 > pH 7.0.

(3) The results from the photolysis stability studies indicated that the solid dispersion technique embedded EM technical in both PVP-K30 and PRIII and afforded a significant advantage for protecting EM from photodegradation. The LC_50_ bioassay results of the 5% wettable powders of the solid dispersions implied that the PVP-K30 solid dispersions furnished improved the solubility and dissolution rate, and also amplified the toxicity of EM against the *P. xylostella* larvae. Moreover, the excellent long-term toxicity of the wettable powder of EM-PRIII solid dispersions against the *P. xylostella* larvae under the field condition was demonstrated, which could prove to be particularly desirable for pest control in comparison to the commercially available emulsifiable 1.5% EM concentrate. These improved performance characteristics could be a result of the sustained-release as well as the reduced photolysis and hydrolysis of EM.

## Figures and Tables

**Table 1:** Dissolution studies of emamectin benzoate (EM) and PVP-K30 with different solvents. Note: The data after “+” indicates the time required for the complete dissolution of EM or PVP-K30 in the solvents by stirring.

Solvents	Solute	Dissolution Time (s)
Methanol	EM	60 + 52 (±9)
	PVP-K30	157 + 0 (±5)
Ethanol	EM	60 + 115 (±11)
	PVP-K30	300 + 67 (±6)
Dichloromethane	EM	60 + 67 (±6)
	PVP-K30	300 + 37 (±5)

**Table 2:** Assignments of IR bands corresponding to vibrations of EM, PVP-K30, and PRIII.

Samples	Wavenumber (cm^−1^)	Assigment
EM	3400	O–H stretching vibration
	2968	-CH_3_ stretching vibration
	2932	-CH_2_ stretching vibration
	1730	C=O stretching vibration
	1600 and 1554	C=C stretching vibration in benzene rings
	1452 and 1380	C-H in-plane bending vibration (saturated bond)
	1158 and 1118	C=O bending vibration in ester groups
	1000-650	Region of C-H out of plane bending vibration (unsaturated bond)
PVP-K30	3416	N–H stretching vibration
	2956	C–H stretching vibration (saturated bond)
	1654	C=C stretching vibration in alkene bonds
	1462 and1372	C-H in-plane bending vibration (saturated bond)
	1290	O–H in-plane bending vibration
PRIII	3000 and 2948	C–H stretching vibration (saturated bond)
	1726	C=O stretching vibration in ester groups
	1485, 1454 and 1274	C–H in-plane bending vibration (saturated bond)
	962 and 752	C–H in-plane bending vibration (unsaturated bond)

**Table 3:** Aqueous solubility results of EM-PVP-K30 solid dispersions prepared using varied feed ratios. Note: Different lower-case letters (such as a, b, c, d, e, f, g, and h) in the same column indicate significant differences in the aqueous solubility of different samples (Duncan’s tests, *p* ≤ 0.05).

Samples	Aqueous Solubility (×10^−3^ mg/mL)	Times of Solubility Improvement
EM technical	23.36 ± 0.33a	-
1:1 feed ratio EM-PVP-K30 solid dispersions	81.76 ± 0.95b	3.5
1:2 feed ratio EM-PVP-K30 solid dispersions	167.38 ± 2.16e	7.2
1:4 feed ratio EM-PVP-K30 solid dispersions	238.43 ± 1.04g	10.2
1:10 feed ratio EM-PVP-K30 solid dispersions	876.22 ± 4.27h	37.5
1:50 feed ratio EM-PVP-K30 solid dispersions	223.95 ± 2.59f	9.6
1:100 feed ratio EM-PVP-K30 solid dispersions	117.48 ± 1.26d	5.0
1:10 feed ratio physical mixture of EM and PVP-K30	99.79 ± 4.15c	4.3

**Table 4:** Kinetic equation describing the release rates of EM from EM-PRIII solid dispersions prepared using varied feed ratios. Note: Different lower-case letters (such as a, b, c, d, and e) in the same column indicate significant differences in the half-life of EM in different samples (Duncan’s tests, *p* ≤ 0.05).

Samples	Kinetic Equation	Equation Type	Correlation Coefficient	Half-life (d)
EM technical	Q = 77.596 (1 − e^−8.672t^)	First-order	0.99	0.12
1:1 feed ratio EM-PRIII solid dispersions	Q = 63.886 (1 − e^−2.284t^)	First-order	0.96	0.67
1:4 feed ratio EM-PRIII solid dispersions	Q = 57.624 (1 − e^−0.842t^)	First-order	0.91	1.81
1:10 feed ratio EM-PRIII solid dispersions	Q = 12.969t^1/2^ + 10.173	Higuchi	0.93	9.43
1:50 feed ratio EM-PRIII solid dispersions	Q = 11.695t^1/2^ + 9.410	Higuchi	0.91	12.05
1:100 feed ratio EM-PRIII solid dispersions	Q = 10.661t^1/2^ + 6.249	Higuchi	0.94	16.76

**Table 5:** Kinetic equation describing the light degradation of EM from EM-PVP-K30 solid dispersions prepared using varied feed ratios. Note: Different lower-case letters (such as a, b, c, d, e, and f) in the same column indicate significant differences in the half-life of EM in different samples (Duncan’s tests, *p* ≤ 0.05).

Samples	Kinetic Equation	Correlation Coefficient	Half-life (h)
EM technical	c_t_ = 88.646e^−0.236t^	0.93	2.42f
1:1 feed ratio EM-PVP-K30 solid dispersions	c_t_ = 87.348e^−0.116t^	0.91	4.81e
1:4 feed ratio EM-PVP-K30 solid dispersions	c_t_ = 89.337e^−0.096t^	0.92	6.07d
1:10 feed ratio EM-PVP-K30 solid dispersions	c_t_ = 89.984e^−0.062t^	0.87	9.43c
1:50 feed ratio EM-PVP-K30 solid dispersions	c_t_ = 93.383e^−0.049t^	0.94	12.67b
1:100 feed ratio EM-PVP-K30 solid dispersions	c_t_ = 93.784e^−0.038t^	0.93	16.47a

**Table 6:** Kinetic equation describing the light degradation of EM from EM-PRIII solid dispersions prepared using varied feed ratios. Note: Different lower-case letters (such as a, b, c, d, e, and f) in the same column indicate significant differences in the half-life of EM in different samples (Duncan’s tests, *p* ≤ 0.05).

Samples	Kinetic Equation	Correlation Coefficient	Half-life (h)
EM technical	c_t_ = 88.646e^−0.236t^	0.93	2.42f
1:1 feed ratio EM-PRIII solid dispersions	c_t_ = 85.096e^−0.131t^	0.87	4.07e
1:4 feed ratio EM-PRIII solid dispersions	c_t_ = 86.891e^−0.099t^	0.89	5.55d
1:10 feed ratio EM-PRIII solid dispersions	c_t_ = 89.320e^−0.082t^	0.91	7.05c
1:50 feed ratio EM-PRIII solid dispersions	c_t_ = 94.038e^−0.053t^	0.96	11.95b
1:100 feed ratio EM-PRIII solid dispersions	c_t_ = 95.997e^−0.033t^	0.96	15.98a

**Table 7:** Bioactivity comparison of different formulations against third-instar larvae of *P. xylostella* (24 h).

Formulation	Toxicity Regression Equation	P Value	LC_50_ (95% CL) (μg/mL)
EM technical	y = 1.000x − 1.362	0.997	23.0 (16.3 − 33.9)
wettable powder of EM-PVP-K30 solid dispersions	y =1.316x − 1.434	0.993	12.3 (9.08 − 16.1)
wettable powder of EM-PRⅢ solid dispersions	y = 0.858x − 1.283	0.996	31.3 (21.0 − 53.3)

**Table 8:** Sustained-release effect of the wettable powder of 5% EM-PRIII solid dispersions. Note: Different lower-case letters (such as a, b, c, d, and e) indicate significantly different pest mortalities between insecticide preparations with varying durations of exposure to the natural environment (Duncan’s tests, *p* ≤ 0.05). Different capital letters (such as A and B) indicate significantly different pest mortalities between two preparations that underwent the same duration of exposure to the natural environment (*t*-test [29], *p* ≤ 0.05).

Days after Spraying (d)	Mortality (%) (48h)	
	Wettable Powder of 5 % EM-PRIII Solid Dispersions	1.5% EM Emulsifiable Concentrate
0	83.19 ± 3.39aA	94.69 ± 4.57aB
5	70.83 ± 5.69bA	74.17 ± 4.19bA
10	58.33 ± 4.30cA	31.67 ± 4.30cB
15	46.67 ± 2.72dA	14.17 ± 3.19dB
20	33.33 ± 4.72eA	5.00 ± 1.92eB
